# Prevalence of stress and associated factors among nursing students in Limpopo province, South Africa

**DOI:** 10.4102/curationis.v49i1.2841

**Published:** 2026-04-29

**Authors:** Ngudo Mangale, Arehone P. Mbada, Mmathabo N. Mothoa, Maite V. Rampedi, Tshepo A. Ntho

**Affiliations:** 1 Department of Nursing Science, Faculty of Health Science, University of Limpopo, Polokwane, South Africa

**Keywords:** stress, undergraduate, nursing, students, mental health

## Abstract

**Background:**

Mental health is a fundamental component of overall well-being and a critical determinant of academic success and professional functioning. However, stress is an often-overlooked burden for nursing students, adversely impacting their learning, clinical performance and overall quality of life.

**Objectives:**

To determine the levels of stress among nursing students and to examine the associations among demographic characteristics. The study was conducted at a selected Nursing Education Institution in Limpopo province, South Africa.

**Method:**

A descriptive, cross-sectional quantitative design was employed to determine stress levels among 262 nursing students. Data were collected through an anonymous, self-administered online questionnaire using the Student Stress Inventory, which demonstrated strong internal consistency (Cronbach’s *α* = 0.910). Data were analysed using descriptive statistics in the Statistical Package for the Social Sciences, Version 30.

**Results:**

Respondents had a mean age of 20.20 years (s.d. = 1.48) and reported moderate stress levels. Female nursing students were significantly more likely to report higher stress than males (*p* < 0.001). Stress levels also differed significantly across study levels (*p* = 0.005), with second-year students reporting the highest stress.

**Conclusion:**

Nursing students face significant stress, which may impact their learning and professional development. This highlights the importance of implementing supportive strategies to foster resilience and coping skills.

**Contribution:**

The study provides evidence to guide targeted interventions promoting students’ mental well-being and academic success.

## Introduction

Mental health conditions such as stress, though often seen as an inevitable part of life, are a hidden burden among nursing students. Rather than being a normal developmental experience, stress among nursing students reflects broader academic, clinical and health system pressures that require deliberate institutional and policy attention (Engelbrecht 2022; Langtree, Razak & Haffejee 2018). Nursing is recognised as one of the most demanding professions, requiring both academic and clinical proficiency. As a result, nursing students often experience levels of stress which significantly impact their academic performance and clinical competence (Alatawi, Morsy & Sharif 2022; Zheng, Jiao & Hao 2022). Stress is defined as the physiological or psychological response to stressors, potentially leading to emotional distress and impaired functioning (American Psychiatric Association [APA] 2013). Symptoms of stress include irritability, fatigue, difficulty concentrating and sleep disturbances. These symptoms are particularly concerning in nursing education, where sustained cognitive focus, emotional regulation and sound clinical judgement are essential for safe patient care.

Stress among nursing students is especially problematic, as it may hinder their ability to meet the rigorous demands of their education and ultimately affect their capacity to provide safe and effective patient care (Labrague 2024). Beyond individual outcomes, stress among nursing students has broader implications for the sustainability of the nursing workforce and the quality of health services. Stress within this population also impacts the United Nations Sustainable Development Goals (SDGs), particularly SDG 3, which aims to reduce mental health disorders, and SDG 4, which seeks to ensure inclusive and equitable quality education (Fields et al. 2021). Addressing stress within nursing education is therefore a public health and policy priority, rather than solely an academic concern. Nonetheless, examination of the literature does emphasise the effectiveness of stress management interventions. A systematic review by Chaabane et al. (2021) reveals that nursing students use coping strategies, such as problem-focused, emotion-focused and dysfunctional coping. Similarly, Labrague (2024) identifies problem-solving behaviours and maintaining an optimistic outlook as common coping strategies. Afonne, Agbakoba and Nwankwo (2023) emphasise problem-solving as the most widely used strategy. However, much of this literature focuses on coping outcomes without sufficiently interrogating how contextual, cultural and institutional factors shape stress experiences among nursing students.

International studies have also revealed high levels of anxiety and depression among nursing students. In Italy, 46.6% of nursing students exhibit moderate to severe anxiety, while 71.7% experience mild to severe depressive symptoms (Curcio et al. 2024). Similarly, in Saudi Arabia, 65.1% of nursing students reported depression (Alreshidi et al. 2024). Equally, Liu et al. (2022) found moderate stress levels among Chinese nursing students, with the primary stressors being the need for advanced knowledge and practical skills. These findings underscore the global prevalence of psychological distress among nursing students, further emphasising the need for mental health interventions. However, most international studies are conducted in well-resourced or urban academic settings, limiting their applicability to Nursing Education Institution (NEIs) operating in rural or resource-constrained contexts. Importantly, stress can lead to poor academic performance, reduced well-being and compromised patient care (Lavoie-Tremblay et al. 2022).

In Africa, the World Health Organization (2022) estimates that over 116 million individuals are affected by mental health challenges, including stress. Notably, students are not immune to these mental health issues. Within the African and South African contexts, students often face additional socio-economic pressures that may exacerbate stress and limit access to adequate mental health support. For instance, a study conducted among physiotherapy students in Gauteng, South Africa, reveals that perceived stress levels vary, with 57.14% of students reporting low levels of stress, while 42.85% experienced moderate stress (Collins et al. 2025). Similarly, a study at a Health Science University in Gauteng found that 25% of participants exhibited symptoms of perceived stress, with significantly higher levels observed in female students than in their male counterparts (Mahlangu, Mokoena & Ntuli 2024). In KwaZulu-Natal, a study underlines that students from diverse socio-economic backgrounds experience depression (Zondi & Santa Mthembu 2023). While informative, these studies are largely urban-based and include mixed health sciences populations, offering limited insight into nursing-specific stressors in rural settings.

A qualitative study in the Limpopo province by Zondi (2018) further identifies elevated stress levels among university students. Limpopo province presents a distinct rural and socio-economically challenged context, where limited institutional resources and support services may intensify students’ stress experiences (Nchabeleng et al. 2024). Additionally, a study in the Western Cape reveals that 72.2% of nursing students reported experiencing moderate stress, with professional education, communication and practical tasks noted as the primary stressors (Mafilika 2022). However, there remains limited evidence focusing on perceived stress among nursing students in Limpopo province, South Africa. Thus, this study aims to determine the perceived stress among nursing students in the selected NEI in Limpopo province. By situating stress within its cultural, educational, and health system context, the findings will contribute to the existing literature, aid interventions and guide policies supporting stress management in NEIs.

### Objective of this study

To determine the levels of stress among nursing students and to examine the associations among demographic characteristics.

### Theoretical framework

This study was guided by Lazarus and Folkman’s Transactional Model of Stress and Coping (1984), which conceptualises stress as a dynamic process arising when individuals perceive that the demands of a situation exceed their available coping resources. Stress is determined not solely by external demands but by the interaction between individuals and their environments (Kromoser 2025; Labrague 2024). This includes their cognitive appraisal of the situation and their available coping strategies. Primary appraisal involves evaluating whether a situation is threatening, challenging or benign; secondary appraisal assesses perceived ability to manage the stressor (Lazarus & Folkman 1984). Coping strategies are then employed to mediate stress, either through problem-focused methods, addressing the source of stress or emotion-focused approaches, regulating emotional responses.

In the context of nursing education, the Transactional Model is relevant, as students at different levels of training face distinct stressors (Lazarus & Folkman 1984). First-year students primarily contend with basic clinical skills and adjustment to the academic environment, reflected in physical and academic stressors (Langtree et al. 2018; Zondi 2018). Second-year students experience heightened academic stressors because of more complex theoretical modules, such as physiology, and the introduction of community-based clinical placements (Mhlongo & Masango 2020; Mudaly 2023). Third- and fourth-year students face advanced clinical responsibilities, preparation for professional integration and cumulative academic pressures, which may increase exposure to environmental and interpersonal stressors.

The Student Stress Inventory (SSI) aligns with this multidimensional view of stress, assessing four interrelated domains (Arip et al. 2020). These domains include physical, academic, interpersonal and environmental stressors, capturing the broad spectrum of stress experiences across all levels of nursing students (Arip et al. 2020). Notably, physical stressors encompass symptoms such as fatigue, headaches and sleep disturbances, reflecting the physiological manifestation of perceived academic and clinical demands (Arip et al. 2020). Academic stressors involve challenges in mastering lessons, meeting deadlines and managing study-related pressures (Arip et al. 2020). Interpersonal stressors include relational challenges with family, peers and faculty, while environmental stressors reflect contextual difficulties such as transportation, campus facilities and residential conditions (Arip et al. 2020). By mapping these sub-scales onto the Transactional Model of Stress and Coping, the study interprets stress as arising from the interaction of perceived demands and coping resources, highlighting why students at different levels may experience different types of stress of varying intensity (Lazarus & Folkman 1984).

Overall, the integration of the Transactional Model with the SSI provides a theoretical lens to understand the multidimensional and cumulative nature of stress in nursing education (Arip et al. 2020; Lazarus & Folkman 1984). It underscores the importance of interventions that target both the demands of the educational environment and the enhancement of students’ coping resources to promote resilience, well-being and academic success across all levels of training.

## Research methods and design

### Study design

A quantitative, descriptive, cross-sectional study was conducted among students at a selected NEI in Limpopo province, South Africa (Botma et al. 2022). A cross-sectional design is appropriate for this study as it allows the determination of stress levels and the examination of associations between stress and selected demographic characteristics at a single point in time, providing a snapshot of the prevalence and distribution of stress among nursing students. While this design permits the identification of statistical associations, it does not allow for causal inference. Given that perceived stress is inherently subjective, a self-report approach was appropriate for capturing students’ experiences. The study was reported in accordance with the Strengthening the Reporting of Observational Studies in Epidemiology (STROBE) guidelines (see Appendix) (Von Elm et al. 2014).

### Study population and setting

The study was conducted at a selected NEI in the Limpopo province of South Africa. The NEI is situated in the Capricorn District, approximately 32 km east of Polokwane, the provincial capital. It is classified among the eight rural and historically disadvantaged institutions in the country and is accredited by the South African Nursing Council (SANC) to train learner nurses under Regulation 174 (R174). The institution offers both theoretical instruction in classrooms and work-integrated learning at accredited clinical practice sites. The study population comprised all undergraduate students enrolled in the Bachelor of Nursing (R174) programme during the 2025 academic year, with a total of 320 students registered across all four levels of study. All registered students who provided informed consent were eligible for inclusion. Students who were not formally registered, those on academic leave of absence or those who declined or withdrew consent were excluded.

### Sampling technique and sample size

The study included all undergraduate students registered in the Bachelor of Nursing (R174) programme during the 2025 academic year, totalling 320 students across the four levels of study. Stratified random sampling was employed to select participants proportionally from each level, ensuring representativeness and minimising sampling bias. The sample size for each level was determined using the Raosoft online calculator, applying a 95% confidence level, a 5% margin of error, and an assumed response distribution of 50%, with a 10% adjustment added to account for potential non-response or incomplete questionnaires. A total of 262 students participated in the study, with each level achieving a high proportional representation of the students selected. Stress was assessed using the SSI, a validated self-report instrument, which captures students’ perceived stress levels at a single point in time.

### Data collection

Data was collected from 18 August 2025 to 05 September 2025. Recruitment was conducted through the distribution of leaflets that outlined the study’s objectives, eligibility criteria and essential contact information. The leaflets invited students to take part voluntarily. Permission to use the SSI was obtained from Arip et al. (2020). For data collection, the SSI questionnaire was uploaded as a Google Form for convenient access, and the link was shared with students via their respective WhatsApp groups. The first page of the form contained an informed consent statement, requiring participants to indicate their consent before accessing the questionnaire. Although Google Forms offers an option to require completion of all items before submission, this feature was not enabled. Given the sensitive nature of stress, participants were allowed to skip any items they felt uncomfortable answering. This approach respected participants’ autonomy and ensured that they could provide responses voluntarily; incomplete responses were monitored for data quality.

The SSI consists of 40 items assessing four domains of stress: Physical Stressors (10 items), Academic Stressors (10 items), Interpersonal Relationships (10 items and Environmental Stressors (10 items). Items were rated on a 4-point Likert scale as follows: Never (1), Somewhat Frequent (2), Frequent (3) and Always (4). The average completion time was 15–20 min. The instrument has demonstrated strong psychometric properties, with a reported content validity of 80.5% and a reliability coefficient of 0.85. Responses were summed to generate both domain-specific and overall stress scores. Sub-scale scores between 10 and 18 indicated low stress, 19–29 moderate stress and 30–40 severe stress. Overall stress levels were classified as follows: 40–80 (mild stress), 81–121 (moderate stress) and 122–160 (severe stress).

### Data analysis

Data analysis for this study involved systematic coding, editing, tabulation and statistical evaluation to summarise and interpret the information collected. The Statistical Package for the Social Sciences (IBM SPSS Statistics, Version 30, IBM Corp., Armonk, New York, United States) was used to perform all statistical analyses. At the same time, Microsoft Excel was employed to organise data and present findings through graphical representations, such as bar charts and pie charts. Descriptive statistics, including frequencies and percentages, were used to provide an overview of stress levels among nursing students, categorising participants into mild, moderate or severe stress groups. Associations between demographic characteristics and the four stressor domains of the SSI (physical, interpersonal, academic and environmental) were assessed using Fisher’s Exact Test. Pearson’s correlation coefficients were computed to examine linear relationships between age and each stress domain, as well as interrelationships among the stress domains themselves. To evaluate the influence of gender on overall stress levels, an ordinal logistic regression analysis was conducted. Finally, the Kruskal–Wallis H test was performed to determine whether stress levels differed significantly across students at different levels of study.

### Ethical considerations

The Turfloop Research Ethics Committee granted ethical approval for the study (TREC/654/2025:UG). Permission to conduct the study was also obtained from the Director of the School of Health Care Sciences and the Head of the Department of Nursing Sciences. Participation was entirely voluntary, and individuals provided electronic informed consent prior to completing the survey. All responses were anonymous, and data were stored securely to ensure confidentiality. While the questionnaire inquired about stress levels, which may have prompted participants to reflect on personal stressors, measures were taken to minimise any potential distress. Participants were provided with contact information for the institutional student counselling departments, which are responsible for providing mental health services to students. They were encouraged to seek support from these services if completion of the questionnaire triggered any emotional discomfort.

## Results

A total of 262 undergraduate nursing students participated in the study. The mean age of participants was 20.20 (s.d. = 1.48), with the majority falling between 19 and 21, indicating that the study population was predominantly young adults. Most participants were female (78.6%), and almost all (99.6%) identified as black (African) people, reflecting the gender distribution commonly observed in the nursing profession, which is female-dominated. This also reflects the demographic profile of the selected NEI, a historically disadvantaged institution serving predominantly students from surrounding rural areas. With respect to the level of study, the largest proportion of students was enrolled in Level 2 (27.5%), followed by Level 3 (26.3%), Level 1 (24.8%), and Level 4 (21.4%). Regarding relationship status, most participants (65.6%) reported being single, with 34.4% in relationships. Furthermore, most respondents (89.3%) were bursary holders; 10.7% were not funded through a bursary. Table 1 provides a detailed description of the participants’ characteristics.

**TABLE 1:** Demographic details of respondents.

Variables	Frequency	%
**Gender**		
Male	56	21.4
Female	206	78.6
**Age**		
Mean	20.20	-
s.d.	1.48	-
**Level of study**		
Level 1	65	24.8
Level 2	72	27.5
Level 3	69	26.3
Level 4	56	21.4
**Race**		
Black (African) people	261	99.6
Coloured people	1	0.4
**Romantic relationship status**		
Single	172	65.6
In relationship	90	34.4
**Financial assistance**		
Bursary	234	89.3
Not funded	28	10.7

s.d., standard deviation.

In addition, as shown in Annexure Table 1, the results from the SSI suggest that students reported moderate stress, with mean scores clustering in the moderate range (2–3). Physical stress was notable for constant fatigue (*M* = 2.71, s.d. = 1.01) but minimal for difficulty breathing (*M* = 1.24, s.d. = 0.53). Interpersonal stress was highest for parental expectations (*M* = 3.27, s.d. = 1.09) and lowest for overprotection (*M* = 1.12, s.d. = 0.41). Academic stress was marked by difficult subjects (*M* = 2.88, s.d. = 0.95) and class presentations (*M* = 2.66, s.d. = 1.11), while environmental stress was evident in long lines (*M* = 2.96, s.d. = 1.07) and crowding (*M* = 2.73, s.d. = 1.15). Overall, fatigue, academic demands and environmental discomfort emerged as the primary sources of stress among students.

### Overall self-reported stress level per sub-scale

As shown in Table 2, nursing students were assessed for stress across four domains: physical, interpersonal relationships, academic and environmental. For physical stress, nearly half of the participants reported moderate stress (50.4%), followed closely by mild stress (46.6%), with only 3.1% experiencing severe stress. In the interpersonal relationship domain, most students reported mild stress (55.7%), 42.2% reported moderate stress, and 1.9% reported severe stress. Regarding academic stress, more than half of the participants (55.0%) experienced moderate stress, 25.6% reported severe stress, and 19.5% reported mild stress. Similarly, in the environmental domain, stress was predominantly moderate (54.6%), followed by severe (24.0%) and mild (21.4%). Overall, while moderate and severe stress levels were most prevalent in the academic and environmental domains, mild stress was observed in all domains, particularly physical and interpersonal stressors.

**TABLE 2:** Overall self-reported stress level per sub-scale (*N* = 262).

Variables	Mild stress		Moderate stress		Severe stress	
	*n*	%	*n*	%	*n*	%
Physical	122	46.6	132	50.4	8	3.1
Interpersonal *R*	146	55.7	111	42.2	5	1.9
Academic	51	19.5	144	55.0	67	25.6
Environment	56	21.4	143	54.6	63	24.0

Interpersonal *R*, interpersonal relationship.

### Association among demographics with sub-scales of the student stress inventory

The findings of this study revealed several statistically significant associations, as shown in Table 3. Gender is significantly associated with physical stressors (*p* < 0.001) and academic stressors (*p* = 0.007), suggesting that male and female students experience these domains differently. No significant associations were found between gender and interpersonal relationship stressors (*p* = 0.950) or environmental stressors (*p* = 0.315). The level of study is significantly associated with physical stressors (*p* = 0.011) and academic stressors (*p* = 0.001), indicating that stress experiences vary depending on the stage of study. No significant associations were observed with interpersonal (*p* = 0.166) or environmental stressors (*p* = 0.190). Financial Status is also significantly associated with academic stressors (*p* = 0.015). However, no significant relationships were found with physical (*p* = 0.195), interpersonal (*p* = 0.502) or environmental stressors (*p* = 0.313). In addition, race and relationship status showed no significant associations with any of the stressor domains (all *p* > 0.05).

**TABLE 3:** Association among demographic characteristics and sub-scales.

Characteristics	Physical	Interpersonal *R*	Academic	Environment
Gender	< 0.001*	0.950	0.007*	0.315
Level of study	0.011*	0.166	< 0.001*	0.190
Race	1.000	0.443	0.450	1.000
Relationship status	0.970	0.767	0.541	0.266
Financial assistance	0.195	0.502	0.015*	0.313

Note: Fisher’s Exact Test was used.

Interpersonal *R*, interpersonal relationship.

* , statistically significant (*p* < 0.01).

### Correlation between the age of the participants and the source of stress

A correlation analysis was conducted to examine the relationships between age and stress across the different domains (physical, interpersonal, academic and environmental) as depicted in Table 4. Age showed no significant association with any of the stress domains, indicating that age was not related to stress levels in this sample. In contrast, significant positive correlations were observed among the stress domains. Physical stress was moderately correlated with interpersonal (*r* = 0.408, *p* < 0.001), academic (*r* = 0.337, *p* < 0.001) and environmental stress (*r* = 0.367, *p* < 0.001). Similarly, interpersonal stress was positively associated with academic (*r* = 0.319, *p* < 0.001) and environmental stress (*r* = 0.342, *p* < 0.001), while academic stress was moderately correlated with environmental stress (*r* = 0.368, *p* < 0.001).

**TABLE 4:** Correlation between the age of the respondents and the source of stress.

Items	Variables	1	2	3	4	5
1	Age	1	−0.015	−0.075	−0.004	−0.018
2	Physical	−0.015	1.000	0.408*	0.337*	0.367*
3	Interpersonal relationship	−0.075	0.408*	1.000	0.319*	0.342*
4	Academic	−0.004	0.337*	0.319*	1.000	0.368*
5	Environment	−0.018	0.367*	0.342*	0.368*	1.000

* , statistically significant (*p* < 0.01).

### Logistic regression assessing the association between gender and stress

The final model demonstrated a significantly better fit than the intercept-only model, *χ*^2^(1) = 17.45, *p* < 0.001 as indicated in Table 5. Model fit indices suggest a good fit to the data, Pearson *χ*^2^(1) = 0.86, *p* = 0.353; Deviance *χ*^2^(1) = 0.65, *p* = 0.419. The assumption of proportional odds was met, *χ*^2^(1) = 0.65, *p* = 0.419. Therefore, gender significantly predicts stress levels. Specifically, males have significantly lower odds of reporting higher levels of stress than females (*B* = –1.28, s.e. = 0.31, Wald *χ*^2^(1) = 16.83, *p* < 0.001, 95% CI [–1.90, –0.67]). The odds ratio indicates that males were about 72% less likely to report higher stress levels than females (OR = 0.28, 95% CI [0.15, 0.51]). Observed and expected counts were closely aligned across categories, with Pearson residuals all < 1, further supporting model adequacy.

**TABLE 5:** Logistic regression assessing the association between gender and stress levels.

Predictor	*B*	s.e.	Wald	*df*	*p*-value	OR	95% CI
Gender (male vs. female)	−1.28	0.31	16.83	1	< 0.001	0.28	0.15, 0.51

*B*, regression coefficient; s.e., standard error; Wald, Wald chi-square test; *df*, degrees of freedom; *p*-value, probability value; OR, odds ratio; 95% CI, 95% confidence interval for the odds ratio.

### Differences in stress levels across levels of study

The results indicate a statistically significant difference in stress among study levels, *H*(3) = 12.71, *p* = 0.005. As shown in Table 6, Level 2 students reported the highest mean rank of stress (MR = 152.47), while Level 3 students had the lowest mean rank (MR = 114.25). Level 1 (MR = 127.34) and Level 4 (MR = 130.63) reported intermediate stress levels. Post-hoc pairwise comparisons using Bonferroni adjustment reveal that Level 2 students experience significantly higher stress than Level 3 students (*p* = 0.003). No other pairwise comparisons reached statistical significance. These findings suggest that stress levels vary across study years, with second-year students reporting the greatest stress.

**TABLE 6:** Post-hoc pairwise comparisons (Bonferroni-adjusted).

Comparison	MR G1	MR G2	TS	std. TS	*p*-value (Unadj.)	*p*-value (Adj.)
Level 2 – Level 3	152.47	114.25	38.21	3.50	< 0.001	0.003*
Level 1 – Level 2	127.34	152.47	−25.13	−2.27	0.023	0.140
Level 4 – Level 2	130.63	152.47	21.84	1.89	0.058	0.351
Level 3 – Level 1	114.25	127.34	13.09	1.17	0.243	1.000
Level 3 – Level 4	114.25	130.63	−16.37	−1.41	0.160	0.960
Level 1 – Level 4	127.34	130.63	−3.29	−0.28	0.781	1.000

MR G1, mean rank of group 1; MR G2, mean rank of group 2; TS, test statistic; std. TS, standardised test statistic; *p*-value (Unadj.), unadjusted significance level; *p*-value (Adj.), Bonferroni-adjusted significance level.

* , statistically significant (*p* < 0.01).

## Discussion

This study aimed to determine the stress levels and factors associated with stress among nursing students in a selected NEI in Limpopo province. The findings reveal that most nursing students experience a moderate level of stress (50.4%) and only 3.1% perceived a severe level of stress. Compared with findings from previous studies conducted among students in other professions and faculties in South African Higher Education Institutions, the level of stress reported in this study appears higher (Collins et al. 2025; Mahlangu et al. 2024). This difference may be attributed to the unique demands and complexities of nursing education, which requires students to balance heavy theoretical workloads with emotionally taxing work-integrated learning. In addition, nursing students are frequently exposed to situations involving human suffering, death and ethical dilemmas, which may compound their stress levels compared to students in other health science disciplines and faculties (Langtree et al. 2018; Nchabeleng et al. 2024).

The level of stress observed in this study aligns with reports from other countries like the Middle East and North Africa, Saudi Arabia and Spain, where moderate stress levels among nursing students are commonly reported (Ahmed & Mohammed 2019; Chaabane et al. 2021; Onieva-Zafra et al. 2020). However, exact stress levels vary across contexts as a result of differences in educational settings, clinical exposure, cultural factors and study methodologies (Alkharj et al. 2024; Cheng et al. 2023; Efstathiou et al. 2025; Labrague 2024). These findings underscore that, although stress is a widespread concern for nursing students, contextual factors must be considered when designing interventions to support their well-being. In addition, factors such as cultural differences in responding to mental health surveys and the use of different stress scales may also influence reported stress levels, underpinning the need for context-specific approaches to assessment and support.

The findings of this study indicate that female nursing students are more likely than their male counterparts to experience higher levels of physical and academic stress. This pattern is consistent with previous studies suggesting that females experience higher levels of emotional and psychological strain and are more likely than males to acknowledge and report these experiences in surveys (Alharbi et al. 2025; Manana et al. 2023; Mutinta 2022). Such gender differences may reflect socio-cultural expectations and gendered roles, where female students often navigate academic pressures alongside additional responsibilities in their personal and social lives. Males, on the other hand, may be less likely to acknowledge or report the stress they experience, which might mask underlying difficulties. These dynamics emphasise the importance of developing gender-sensitive strategies within nursing education, aimed at supporting both male and female students in managing stress effectively through tailored coping mechanisms. Providing tailored academic mentorship, promoting adaptive coping mechanisms and fostering supportive learning environments may help mitigate stress and enhance the overall well-being and academic success of female nursing students.

The study findings regarding age align with previous research, which has reported that age does not significantly influence stress levels among nursing students (Aslan & Akturk 2018; Collins et al. 2025). In contrast, the study identifies significant positive correlations among the stress domains (physical, interpersonal, academic and environmental), suggesting that stress in one area may exacerbate stress in others. This aligns with existing literature indicating that stressors in higher education often arise from a combination of academic, social, environmental, physical and psychological factors, which interact to influence students’ overall stress experience (Sharma, Karmakar & Khan 2024; Yikealo, Yemane & Karvinen 2018). From the perspective of Lazarus’ Transactional Model of Stress and Coping (Lazarus & Folkman 1984), these findings can be interpreted as reflecting the cumulative effect of multiple stressors. The interrelated nature of stress domains suggests that interventions aimed at supporting students should address multiple areas simultaneously to enhance overall coping capacity and reduce the cumulative burden of stress. These areas include academic, social, environmental and personal.

The present study indicates that second-year nursing students experience more elevated stress levels than their peers in other academic years. These findings are corroborated by findings from other studies noting that second-year students often encounter increased academic and clinical pressures, which may contribute to heightened stress levels (Admi et al. 2018; Lavoie-Tremblay et al. 2022). Notably, the new nursing education programme R174, as accredited by SANC, is more complex during the first and second years, as nursing students are introduced to more complex academic modules, such as physiology, which many find challenging (Mhlongo & Masango 2020; Mudaly 2023). In contrast, third-year students reported comparatively lower stress levels, suggesting that experience and the development of coping strategies may mitigate some of the pressures associated with nursing education. Consistent with this, research indicates that third- and fourth-year nursing students demonstrate improved organisational skills and have developed strategies to achieve a healthier balance between their academic responsibilities and personal lives (Lavoie-Tremblay et al. 2022). However, despite the potential for stress reduction in later years, nursing students remain a high-stress group because of the nature of their education programme, which combines rigorous theoretical classroom learning with demanding work-integrated clinical training.

The findings of this study reveal a significant association between financial assistance (or funding support) and academic stressors, a relationship consistent with previous research (Mudaly 2023; Ngoc & Tuan 2024; Steenkamp & Chipps 2024; Ullah et al. 2025; Zwane & Mukuna 2023). This underscores the persistent influence of economic constraints on students’ ability to manage academic demands, particularly for those who do not receive funding. This can be understood through the lens of Lazarus’ Transactional Model of Stress and Coping (Lazarus & Folkman 1984), which posits that stress arises when individuals perceive that the demands of a situation exceed their available coping resources. In this context, students with limited financial resources may perceive academic tasks such as meeting tuition obligations, purchasing learning materials or managing other education-related expenses as overwhelming, thereby increasing their experience of academic stress. Collectively, these findings underscore the need for targeted financial and academic support to help students manage the demands of their nursing education effectively.

### Limitations

This study relies on self-reported data, which may be subject to social desirability and recall bias. As a result, the reported stress levels may underestimate or overestimate the true prevalence of stress among nursing students, potentially affecting the accuracy of associations with demographic factors. The cross-sectional design limits causal inferences, providing only a snapshot of stress at a single point in time. Consequently, observed relationships between stress and demographic characteristics are correlational and do not reflect temporal changes or causality. Data were collected from a single NEI, which may limit generalisability to other settings. Thus, findings should be interpreted cautiously when applying them to nursing students in other provinces or NEIs. Finally, while the SSI demonstrated strong reliability and content validity, future studies could consider using tools specifically validated for South African nursing students, such as the Perceived Stress Scale adapted by Engelbrecht (2021), to enhance construct validity and ensure findings are culturally and contextually relevant. Future research could also employ mixed-methods approach, longitudinal designs and multi-site sampling to provide a more comprehensive understanding of stress and its determinants in this population.

### Recommendations

Based on the findings of this study, several recommendations are proposed for nursing students, NEIs, policymakers, curriculum developers and future research. Individual students should adopt effective coping strategies, including time management, mindfulness, peer support and other stress reduction techniques, particularly female and second-year students who reported higher stress levels. Students should also be encouraged to seek professional mental health support and make use of available counselling and mentorship services to manage both academic and clinical stressors.

For NEIs, it is recommended that gender-sensitive mentorship and support programmes should be developed, acknowledging that female students may experience higher levels of physical and academic stress. Furthermore, NEIs should monitor stress levels regularly to identify high-risk groups and intervene early.

Policymakers are encouraged to ensure adequate resources and funding for student mental health support within nursing education, including the implementation of stress management programmes and peer mentorship initiatives. Policies should also address financial constraints, which are associated with increased academic stress, to reduce barriers to student success.

Curriculum development should incorporate mental health literacy, stress management, and resilience training into the nursing programme, while structuring academic modules and clinical placements in a manner that balances rigour with student well-being. Special attention should be given to first- and second-year students who are introduced to complex theoretical and clinical modules, as these cohorts were observed to experience higher stress levels.

## Conclusion

Stress is a significant and prevalent issue among undergraduate nursing students, particularly females and second-year students, and is influenced by factors such as level of study, gender and financial circumstances. Importantly, mild, moderate and severe stress were observed across multiple domains, highlighting the full spectrum of mental health burden among nursing students. These findings underscore the need for targeted, evidence-based support strategies, including stress management programmes, peer mentorship and accessible mental health services, to enhance students’ well-being, resilience and academic success. Nurse educators should integrate structured stress reduction strategies into the curriculum, while policymakers must ensure adequate institutional and provincial resources for mental health support in nursing education. By linking these findings to the Transactional Model of Stress and Coping, the study provides conceptual insight into how students perceive and respond to stress, strengthening its contribution to scholarly understanding. Addressing student stress not only improves learning outcomes and retention but also contributes to the development of competent, mentally healthy nursing professionals. Furthermore, these findings offer a foundation for policy action and institutional planning, ensuring that interventions are contextually appropriate for South African nursing students. Future research could explore longitudinal patterns of stress and evaluate the effectiveness of context-specific, theory-informed interventions tailored to this population.
